# Effects of Ethanolic and Aqueous Extracts of *Garcinia gardneriana* Leaves in an In Vivo Experimental Model Induced by a Hyperlipidic Diet

**DOI:** 10.3390/nu15061308

**Published:** 2023-03-07

**Authors:** Bruna Larissa Spontoni do Espirito Santo, Lidiani Figueiredo Santana, Wilson Hino Kato Junior, Felipe de Oliveira de Araújo, Mariana Bento Tatara, Júlio Croda, Danielle Bogo, Karine de Cássia Freitas, Rita de Cássia Avellaneda Guimarães, Priscila Aiko Hiane, Arnildo Pott, Wander Fernando de Oliveira Filiú, Bernardo Bacelar de Faria, Patrícia de Oliveira Figueiredo, Valter Aragão do Nascimento, Frederico Louveira Ayres, Paulo Roberto Haidamus de Oliveira Bastos

**Affiliations:** 1 Graduate Program in Health and Development in the Central-West Region of Brazil, Federal University of Mato Grosso do Sul-UFMS, Campo Grande 79079-900, Brazil; 2 Graduate of Pharmaceutical Sciences, Federal University of Mato Grosso do Sul-UFMS, Campo Grande 79079-900, Brazil; 3 Graduate of Electrical Engineering, Federal University of Mato Grosso do Sul-UFMS, Campo Grande 79079-900, Brazil; 4 Health Science Research Laboratory, Federal University of Grande Dourados, Dourados 79804-970, Brazil; 5 School of Medicine, Federal University of Mato Grosso do Sul, Campo Grande 79070-900, Brazil; 6 Oswaldo Cruz Foundation, Campo Grande 79074-460, Brazil; 7 Laboratory of Botany, Institute of Biosciences, Federal University of Mato Grosso do Sul, Campo Grande 79070-900, Brazil; 8 Faculty of Pharmaceutical Sciences, Food and Nutrition, Federal University of Mato Grosso do Sul-UFMS, Campo Grande 79079-900, Brazil; 9 Diagnostic Medicine Laboratory-Scapulatempo, Campo Grande 79002-17, Brazil; 10 Laboratory of Pronabio (Bioactive Natural Products), Chemistry Institute, Federal University of Mato Grosso do Sul-UFMS, Campo Grande 79079-900, Brazil; 11 Medical School, Federal University of Mato Grosso do Sul, Campo Grande 79070-900, Brazil

**Keywords:** Brazilian plant, bacupari, medicinal plant, metabolic changes

## Abstract

The study of medicinal plants, such as the genus *Garcinia* (Clusiaceae), in the treatment of non-communicable chronic diseases has aroused the interest of researchers. However, there are no studies in the literature that have investigated the effects of *Garcinia gardneriana* in experimental models of obesity for possible metabolic alterations. Swiss mice receiving a high-fat diet were supplemented with aqueous or ethanolic extract of *G. gardneriana* at doses of 200 or 400 mg/kg/day. It was found that there was a reduction in food consumption in experimental groups compared with the control groups, and the group supplemented with aqueous extract at a dose of 200 mg/kg/daydisplayed a reduction in weight. The results showed an increase in the values of high density lipoprotein (HDL-c), total cholesterol, triglycerides and fasting blood glucose. *G. gardneriana* did not protect against insulin resistance, and caused in an increase in monocyte chemoattractant protein-1 (MCP-1) concentrations and a reduction in interleukin 10 (IL-10). In addition, hepatic steatosis and microvesicular steatosis were indicated. It was revealed that, under the experimental conditions in the study, *G. gardneriana* did not prevent weight gain or comorbidities; that is, a different behavior was obtained from that described in the literature with regard to the medicinal potential of the *Garcinia* species, which is probably related to the phytochemical properties.

## 1. Introduction

Garcinia is a genus of Clusiaceae plants, belonging to the Guttiferae family, comprising approximately 40 genera and 1200 species distributed in regions such as tropical Asia, Africa, New Caledonia, Polynesia and Brazil [1,2]. The Garcinia species are rich in secondary metabolites, such as prenylated and oxygenated xanthones [3], which have antifungal [4], anti-inflammatory [5], antitumor [6], antioxidant [7] and antilipidemic activities [6,7,8,9], as well as human immunodeficiency virus (HIV) inhibitory properties [8].

Native to the Atlantic Forest, *Garcinia gardneriana* (Planchon and Triana) Zappi. (Clusiaceae), or *Rheedia gardneriana*, is a species of the genus Garcinia that is native to the Atlantic Forest, is easily found and is popularly known as “bacupari”, “bacopari”, “bacopari miúdo” or “yellow mangostão” [10]. In different parts of the plant, xanthones, steroids, triterpenes and flavonoids are found, as secondary metabolites that manifest anti-inflammatory, antinociceptive, antibacterial and antiparasitic effects [3,11,12,13,14].

Such a composition can manifest beneficial effects in the prevention and treatment of obesity, which is a chronic, multifactorial disease (triggered by the influence of social, behavioral, environmental, cultural, psychological, metabolic and genetic issues) and its metabolic changes [15]. Furthermore, it is known that being overweight or obese has increased significantly in recent years and affects both adults (27.5%) and children (47.1%) worldwide [16]. In Brazil, 20.3% of the population is obese, and 55.4% are overweight [17].

Obesity is a chronic disease defined as an abnormal or excessive accumulation of fat, representing a risk for health. The excessive accumulation of fat, mainly in the visceral region, is directly related to the expression of several proteins, favors increased expression of tumor necrosis factor alpha (TNF-α), monocyte chemotactic protein (MCP-1/CCL-2) and interleukin-6 (IL-6) by monocytes and macrophages, manifesting a pro-inflammatory effect. It is also directly related to the development of insulin resistance (IR), which is decisive for the risk of developing several diseases, such as: arterial hypertension, dyslipidemia, insulin resistance, type 2 and metabolic syndrome, pathologies that trigger the development of cardiovascular diseases [18].

In order to reduce the incidence and prevalence of obesity and its metabolic disorders, many treatments are proposed. The literature has aroused interest in investigating the use of medicinal plants with active components that reduce inflammatory markers and oxidative stress, as well as prevent hypercholesterolemia and hypertriglyceridemia [19].

In this sense, the leaves of *G. gardneriana* present a chemical profile with the presence of a wide variety of phenolic compounds, especially flavonoids, biflavonoids (GB-2a, Fukugentin and Fukugiside), catechins, xanthones, in addition to benzophenones (7-epiclusanone) [11,20], that demonstrate properties associated with pharmacological effects, such as anti-inflammatory, antinociceptive, antibacterial and antiparasitic action. They have promising components for the prevention and treatment of several diseases [11] and have stimulated the development of the study described here, involving an experimental model where a high-fat diet was consumed [21,22].

To date, there are no studies that show the effect of such properties; moreover, there is no evidence of the possible beneficial effect of *Garcinia gardneriana* (Planchon and Triana) Zappi. (Clusiaceae) [11] against obesity and its metabolic disorders [23,24]. Thus, the objective of this research was to evaluate the therapeutic effects of ethanolic and aqueous extracts of *G. gardneriana* in animals receiving a high-fat diet.

## 2. Materials and Methods

### 2.1. Leaf Collection

The leaves of *G. gardneriana* were obtained in an urban area of Campo Grande (latitude 20.533720 and longitude 54.6751460), State of Mato Grosso do Sul, Brazil. It was registered as number A26D547 in the National System for the Administration of Genetic Heritage and Associated Traditional Knowledge (Sisgen). After collection, the leaves were placed in an air ventilation oven at 40 °C in the laboratory of the Food Science Unit (Unical-UFMS-Campo Grande-MS-Brazil) of the Faculty of Pharmaceutical Sciences, Food and Nutrition (Facfan UFMS-Campo Grande-MS-Brazil) of the Federal University of Mato Grosso do Sul (UFMS), then ground and homogenized in a Turrax™ mixer. It was labelled and conserved in dark packages until the preparation of the extracts [24].

### 2.2. Extract Preparation

To obtain the ethanolic extract, the leaves of *G. gardneriana* were macerated. For each 1 kg of powder, 10 L of ethanol were added and left to rest for 7 days, after which they were filtered. The filtrate was re-extracted with ethanol five more times. For the preparation of the aqueous extract, the leaves were also macerated. For every 1 kg of powdered *G. gardneriana* leaves 10 L of distilled water was added, kept at rest for 1 day, and then filtered. Both were concentrated under reduced pressure at 37 °C and lyophilized [25].

### 2.3. Animals

The experimental protocol was approved by the Ethics Committee for Animal Use (Protocol no 1050/2019), according to the International Guiding Principles for Bio-medical Research Involving Animals (CIOMS), Geneva, 1985; the Universal Declaration of Animal Rights (UNESCO), Brussels, Belgium, 1978; and National Institutes of Health guidelines on the use and care of laboratory animals. 130 male Swiss mice of the Mus musculus lineage, adults with 60 days of age, were used. The mice were kept in collective cages (30 × 20 × 13) (GC100) (four to five animals per box) in the animal experimentation room of the Biotherium of the Federal University of Mato Grosso do Sul, under controlled temperature conditions at 22 ± 2 °C. C, relative air humidity of 50–60% and light/dark cycle of 12/12 h [26].

### 2.4. Experimental Design

**Figure 1:**
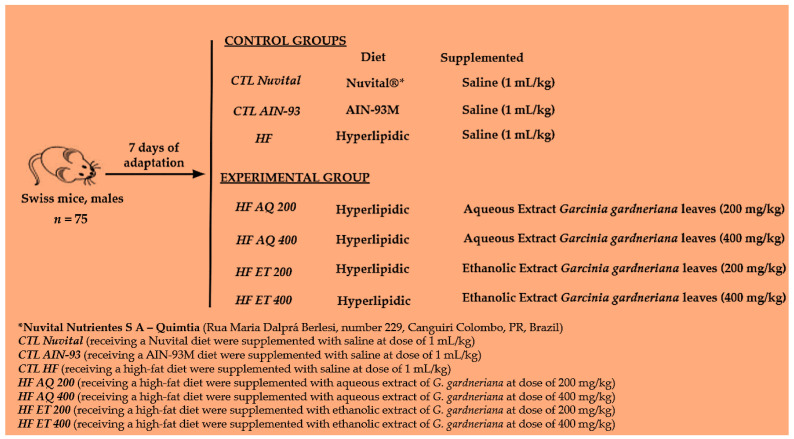
Experimental model design using a high-fat diet.

Swiss mice (male and adult) underwent 7 days of adaptation to the new environment, and were then divided into experimental groups, as shown in Figure 1.

On the first day of the experimental design, the ration was changed simultaneously with the supplementation, which was performed by gavage, in which saline solution (1 mL/kg of animal weight) was administered to the control groups and the aqueous extract to the experimental groups (200 or 400 mg/kg) or ethanolic extract (200 or 400 mg/kg) of *G. gardneriana* leaves (Figure 1). The experiment lasted 8 consecutive weeks [27,28,29]. The doses were defined based on a study that used *Garcinia cambogia*, given, to our knowledge, the absence of in vivo studies with the *G. gardneriana* species [30,31].

**Figure 2:**
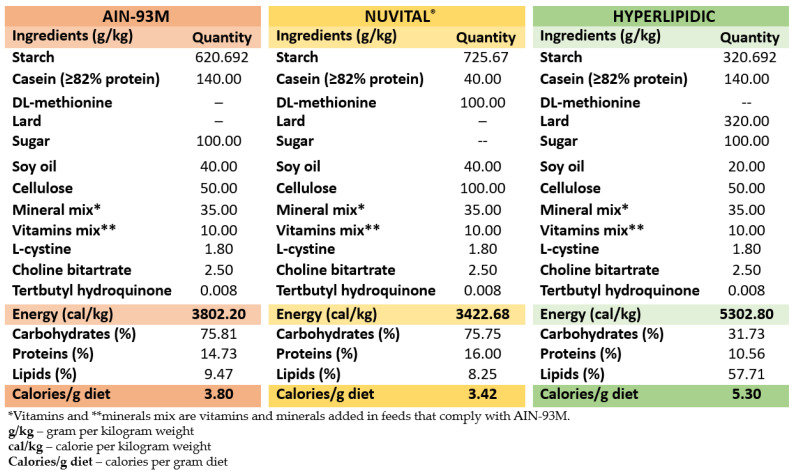
Composition of experimental diets (g per kg of feed).

Each group had ad libitum access to water and food during the experimental period; the high-fat diet used in this study was based on that of Lenquiste et al., 2015 [32] and according to experiences of our research group [33] (Figure 2).

Food consumption was monitored weekly, considering the difference in grams between the amount offered and leftovers (per animal). The animals’ body weights were evaluated weekly on a semi-analytical scale (Bel^®^ São Paulo-Brazil) [34].

After 8 weeks of treatment and 8-h fasting, the animals were anesthetized with Isofluorane^®^ BioChimico^®^-Brazil and euthanized by exsanguination through the inferior vena cava. Blood samples were centrifuged at 3000 rpm for 5 min, and the serum was separated and stored at −18 °C in a biofreezer for further analysis [35].

Adipose tissue was also removed (epididymal, retroperitoneal, perirenal, mesenteric and omental) to determine the animal’s fat content (percentage of adipose tissue at each site in relation to body weight) [35].

### 2.5. Metabolic Changes in Serum

The blood of all animals was collected at the end of the experimental period for serum analysis, and serum samples were used to determine the following parameters: fasting glucose, triglycerides, total cholesterol and fractions High Density Lipoprotein (HDL-c), Low Density Lipoproteins (LDL-c) and Very Low Density Lipoproteins (VLDL-c), evaluated by colorimetric kits (Labtest Diagnostics SA™, Lagoa Santa, MG, Brazil).

The oral glucose tolerance test (OGTT) was also performed, which occurred 4 days before the end of the experiment, for which the animals were weighed after 12 h of fasting, and then the fasting glycemia was checked via caudal (time 0), using a glucometer. Then, the animals received glucose via gavage at a concentration of 2 g/kg of body weight. The glycemia was determined at 15, 30, 60 and 120 min after the administration of glucose [36].

For the insulin sensitivity test (IST), which was performed 2 days before the end of the experiment, the animals were weighed and blood glucose was checked in the fed state (time 0). Then, 1.5 U/kg of insulin (NovoRapid^®^ -São Paulo, SP, Brazil) was applied intraperitoneally, and blood glucose was evaluated at times 0, 15, 30 and 60 min [35].

For both OGTT and IST, blood glucose values were recorded and the area under the curve (AUC) was calculated [36,37].

### 2.6. Concentration of Adipokines: IL-10 and MCP-1

The collected serum was vortexed for 30 s and placed in a centrifuge (5000 rpm for 10 min). Afterwards, 10 µL of serum from each animal, 10 µL of assay buffer and 25 µL of solution containing the adipokines IL-10 and MCP-1 were distributed in a 96-well plate, following the instructions of the commercial kit MAD-KMAG-71K^®^ by Merck-Sigma Aldrich^®^ São Paulo-Brazil, reading the plate on the Luminex^®^ by Merck-Sigma Aldrich^®^ São Paulo-Brazil using the MAGPIX^®^ software by Merck-Sigma Aldrich^®^ São Paulo-Brazil, and the concentration values were obtained in µg/mL.

### 2.7. Statistical Analysis

Statistical analysis was performed using the software Jandel Sigma Stat, version 3.5 (Systat software, Incs., San Jose, CA, USA), and Sigma Plot, version 12.5 (Systat Software Inc., San Jose, CA, USA) and presented as mean ± standard deviation (SD). The groups were compared using one-way ANOVA followed by Tukey’s post test. Differences were considered significant when *p* < 0.05.

## 3. Results

### 3.1. Food Intake, Weigh Again, Body Fat Percentage and Adipocyte Area

**Figure 3:**
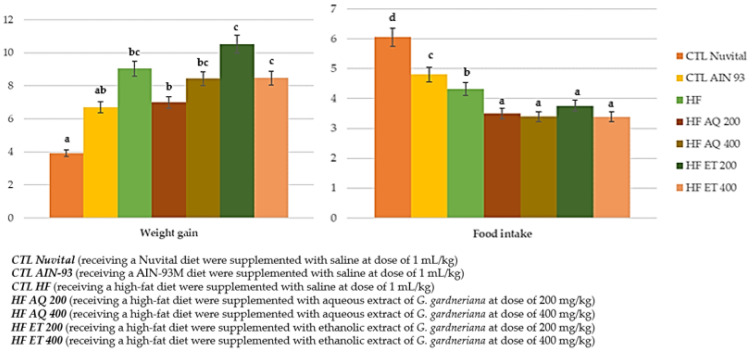
Weight gain (g) and food intake (g) of animals fed a balanced or high-fat diet and supplemented with saline, aqueous extract or ethanol extract of *G. gardneriana* leaves per day for 8 consecutive weeks. Values expressed as mean ± standard deviation. Different letters indicate a significant difference between groups and groups with the same letter are not significantly different. *p* < 0.001; *n* = 6–11; ANOVA followed by Tukey post-test.

During the 8 weeks of supplementation and being fed with high-fat diets, it was observed that the intake of food was significantly lower in all groups of animals supplemented with aqueous or ethanolic extracts independently of the dose compared with the control (CTL) Nuvital, CTL AIN93 and High Fat (HF) groups (*p* < 0.001). However, weight gain was not prevented for the HF AQ 400, HF ET 200 and HF ET 400 groups that received the 200 mg/kg dose. The HFAQ200 group gained less weight than the other groups under the hyperlipidic diet and showed values close to the CTL AIN93 and CTL Nuvital groups (Figure 3).

**Figure 4:**
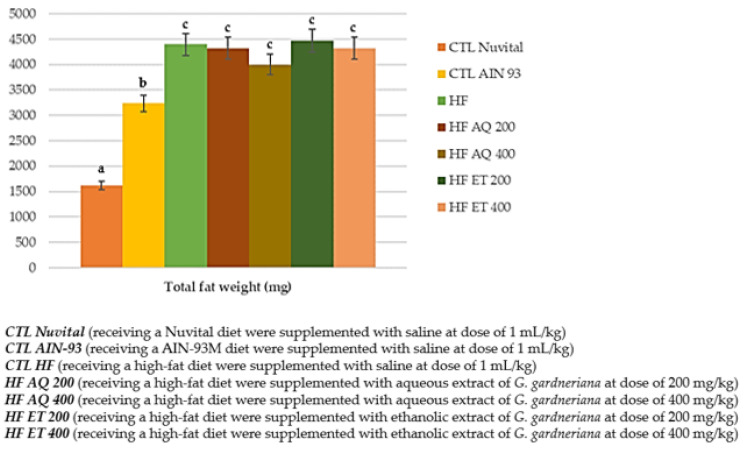
Total fat weight (mg) of animals fed a balanced or high-fat diet and supplemented with saline, aqueous extract or ethanol extract of *G. gardneriana* leaves per day for 8 consecutive weeks. Values expressed as mean ± standard deviation. Different letters indicate a significant difference between groups and groups with the same letter are not significantly different. *p* < 0.001; *n* = 6–11; ANOVA followed by Tukey post-test.

Thus, all evaluated extracts had a reduction of food intake; however, they had no impact on weight gain, with the exception of HF AQ 200. In addition, they did not impact the reduction in adipose tissue accumulation (Figure 4).

### 3.2. Serum Metabolic Changes

#### 3.2.1. Triglycerides and Cholesterol (Total and Fractions) in Serum

**Figure 5:**
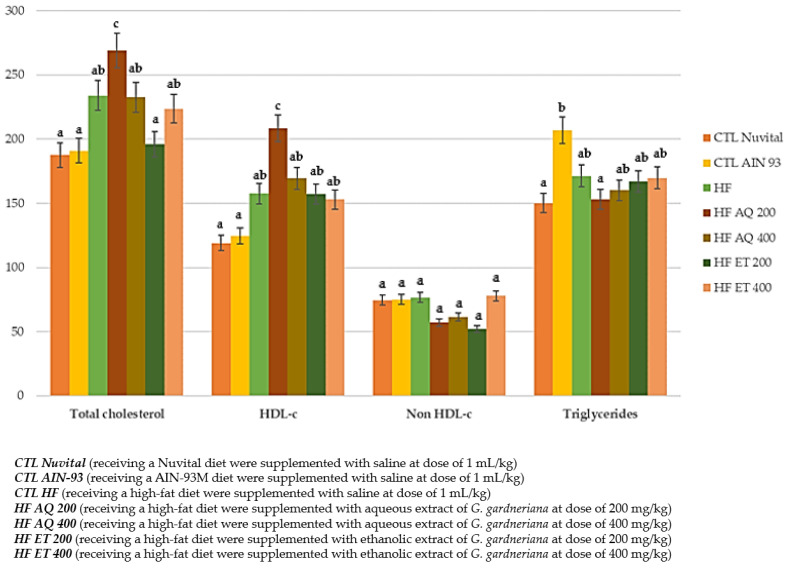
Total cholesterol, fractions and triglycerides (mg/dL) of animals fed a balanced or high-fat diet and supplemented with saline, aqueous extract or ethanol extract of *G. gardneriana* leaves per day for 8 consecutive weeks. Values expressed as mean ± standard deviation. Different letters indicate a significant difference between groups and groups with the same letter are not significantly different. *p* < 0.001; *n* = 6–11; ANOVA followed by Tukey post-test.

The ethanol extract at a dose of 200 mg/kg significantly reduced total cholesterol values (*p* < 0.001), while the other experimental groups showed higher values with a statistical difference compared with the CTL Nuvital, CTL AIN 93 and HF ET 200 groups (Figure 5).

In the HDL-c fraction, it was noted that all groups receiving supplementation with aqueous or ethanolic extracts of *G. gardneriana*, regardless of dose, obtained higher values with a statistical difference compared with the CTL Nuvital and CTL AIN 93 groups, while the HF AQ 200 group showed higher values than all the others (*p* < 0.001). There was no difference among the groups for the non-HDL fraction or triglycerides (Figure 5).

Thus, these results show that the ethanol extract at a dose of 200 mg/kg improved the HDL-cholesterol values even under hyperlipidic diet conditions.

#### 3.2.2. Glycemic Profile: Fasting Blood Glucose, Oral Glucose Tolerance and Insulin Sensitivity Tests

**Figure 6:**
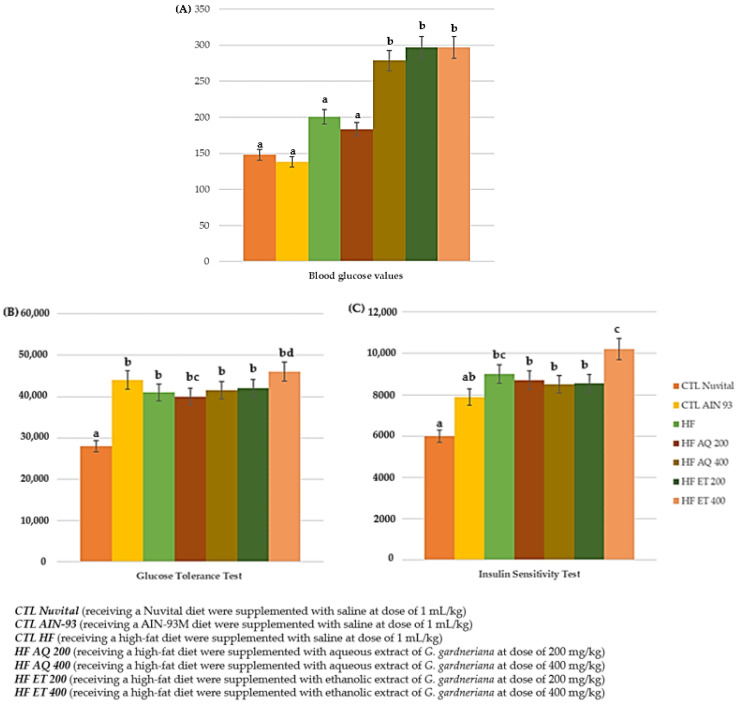
Blood glucose values (mg/dL) (**A**), and the area under the curve of the insulin sensitivity test (%) (**B**) and the oral glucose tolerance test (%) (**C**) of animals fed a balanced or high-fat diet and supplemented with saline, aqueous extract or ethanol extract of *G. gardneriana* leaves per day for 8 consecutive weeks. Values expressed as mean ± standard deviation. Different letters indicate a significant difference between groups and groups with the same letter are not significantly different. *p* < 0.05; *n* = 6–11; ANOVA followed by Tukey post-test.

When evaluating the glycemic profile, it was observed that fasting glucose worsened; that is, there was a significant increase in the HF AQ 400, HF ET 200 and HF ET 400 groups compared with the CTL Nuvital, CTL AIN 93, HF and HF AQ 200 groups (*p* < 0.05) (Figure 6A). In contrast, there was a reduction in the fasting blood glucose values in the HF AQ 200 group compared with the other groups (*p* < 0.05) (Figure 6A). However, there was no impact on the results obtained in the evaluation of the area under the curve of the oral glucose tolerance test or the insulin sensitivity test (Figure 6B,C).

#### 3.2.3. Adipokine Concentration: Cytokines IL-10 and MCP-1

**Figure 7:**
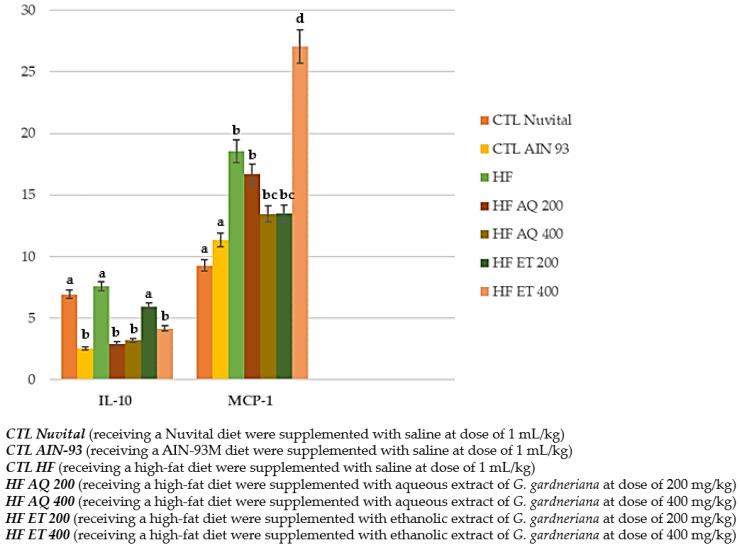
Quantification of IL-10 and MCP-1 (pg/mL) in animals fed a balanced or high-fat diet and supplemented with saline, aqueous extract or ethanol extract of *G. gardneriana* leaves per day for 8 consecutive weeks. Values expressed as mean ± standard deviation. Different letters indicate a significant difference between groups and groups with the same letter are not significantly different. *p* < 0.001; *n* = 6–11; ANOVA followed by Tukey post-test.

In the quantification of the cytokine interleukin 10 (IL-10), it was observed that the CTL AIN 93, HF AQ 200, HF AQ 400 and HF ET 400 groups presented lower values with a statistical difference compared to the CTL Nuvital, HF and HF ET 200 groups (*p* < 0.001). This is in contrast to the MCP-1 quantification, in which all groups supplemented with *G. gardneriana* presented much higher values with a statistical difference compared with the control groups (*p* < 0.001) (Figure 7).

Therefore, this shows that the HF AQ 200, HF AQ 400 and HF ET 400 groups had reduced IL-10 values, but there was no effect on MCP-1 concentrations.

## 4. Discussion

The development of research that investigates the phytochemical composition and therapeutic properties of medicinal plants enables innovation for the production of medicines and herbal isolates, whose nutritional composition of micronutrients and secondary metabolites determines the nutraceutical effects and their medicinal functions [38,39]. They can be promising candidates for the treatment of obesity when compounds are present that manifest effects in the prevention and treatment of obesity and the possible metabolic alterations, such as through reduction of body fat and/or improvement of the serum lipid profile, which can protect against metabolic alterations [40,41].

In this context, numerous phytochemical and biological studies on Garcinia species have been carried out to date, validating their traditional functions under a modern scientific perspective and developing their new pharmacological activities. The extracts of this genus are rich in polyprenylated polycyclic acylfloroglucinols (PPAPs), xanthones, polyphenols and flavonoids [11,12], being described in the literature as healing agents with anti-inflammatory, antioxidant, antitumor, antifungal, anticancer, antihistamine, antiulcerogenic, antimicrobial, antiviral, vasodilator, hypolipidemic, hepatoprotective, nephroprotective and cardioprotective properties [41]. Garcinia species are widely studied for their possible effects on weight loss, due to the presence of compounds that favor fat burning and satiety suppression [21,22].

The leaves of *G. gardneriana* present sitosterol and stigmasterol, α-copaene, α-muurolene, γ-cadinene and cadinene, which are phytosterols and sesquiterpenes, which have already been identified as potent anti-inflammatory and anticancer agents [42]. *G. gardneriana* also contains xanthones, steroids, triterpenes and flavonoids, secondary metabolites that have been associated with anti-inflammatory, antinociceptive, antibacterial and antiparasitic activities [10,11,12,14,20]; however, the present study is the first to investigate the effects of *G. gardneriana* on an experimental model with high-fat diets.

Therefore, in this study, after 8 weeks of supplementation of a high-fat diet with aqueous extract or ethanolic extract of *G. gardneriana* leaves (doses of 200 or 400 mg/kg), there was a significant reduction in food consumption; however, only the aqueous extract in the 200 mg/kg dose reduced weight gain without influencing adiposity. This finding is contrary to those in the study by Lim et al. (2020) [43], in which, after 9 weeks of treatment, obese rats treated with methanolic extract of *Garcinia atroviridis* pulp at doses of 100, 200 and 400 mg/kg had a lower food intake and consequently lower weight gain, manifesting a slimming effect.

The same condition was observed in the study by Muhamad Adyab et al. (2019) [44], who investigated the potential of aqueous extract of *Garcinia mangostana* pulp on metabolic and structural changes in obese rats induced by a hyperlipidic diet (diet providing 414.0 kcal/100 g of energy with 43% carbohydrate, 17% protein and 40% fat), supplemented for 7 weeks at different doses (200, 400 and 600 mg/kg); a significant reduction in food intake, body weight and adiposity was observed.

Our data, therefore, provide us with the fact that supplementation with aqueous extract or ethanolic extract of *G. gardneriana* in mice fed a hyperlipidic diet reduced the amount of food consumed and subsequently suppressed excessive body weight gain. These observations support the hypothesis that *G. gardneriana* has weight-reducing properties.

Chae et al. (2016) [45], who investigated the effects of *Garcinia mangostana* on metabolic syndrome in mice fed with a high-fat diet and the underlying mechanisms related to adipogenesis, also observed a lower food intake and body weight, as well as suppressed adiposity, in the groups supplemented with ethanolic extract of *Garcinia mangostana* bark at 50 or 200 mg/kg doses.

Such results are attributed to the fact that the species *Garcinia mangostana* and *Garcinia atroviridis* contain the main compound hydroxycitric acid, which contributes to antioxidant activity and is believed to induce weight loss by reducing the lipogenesis process and by appetite suppression [46]; furthermore, low values of hydroxycitric acid lactone are found in *G. gardneriana* [11]. These studies also pointed to a possible effect on sensitive protein kinases for the regulation of intracellular energy (active protein kinase—AMPK), thus influencing the intracellular energy balance, and consequently impacting the energy metabolism of the whole organism; this effect could play a protective role against metabolic diseases such as obesity [47], as well as being an important regulator of lipid and glycemic metabolism [48].

In this sense, in the present study, a significant reduction in total cholesterol values was observed in the group supplemented with ethanolic extract of *Garcinia mangostana* bark at doses of 50 or 200 mg/kg (ethanolic extract), along with an increase in HDL-c values in all groups supplemented with either aqueous or ethanolic extract of *G. gardneriana* independent of dose, but with increased concentrations of the non-HDL fraction and triglycerides, results that are in agreement with other studies [43,44,45]. This finding can be attributed to evidence already published in other studies, such as Patil (2015) [49] and Demenciano et al. (2020) [11], who identified the presence of xanthones, flavonoids and catechins, which have antioxidant properties similar to that of vitamin C (ascorbic acid) that help to inhibit the action of free radicals and are related to lipid peroxidation, thus regulating the serum lipid profile. In addition, xanthones have been shown in other studies to play a role in the normalization of metabolic abnormalities and exert good pharmacological effects in animal models of metabolic syndromes, diabetes and obesity, having cardioprotective, antioxidant, anti-inflammatory and anti-obesity agents [44].

The consumption of high-fat diets has been shown to contribute to the development of hyperlipidemia and glucose intolerance [50]. In the present study, hyperglycemia was demonstrated in the groups supplemented with ethanolic or aqueous extract of *G. gardneriana*, regardless of dose. The results of the HF AQ 200 group indicated reduced fasting blood glucose values compared with the other groups; the results obtained for the oral glucose tolerance test and insulin sensitivity test indicated an increase in the area under the curve values in the same groups, while the HF AQ 200 group failed to maintain the protection demonstrated in the fasting glucose test.

Evidence in the literature confirms that high-fat diets result in disturbances in glucose metabolism and glucose intolerance [49], thus causing insulin sensitivity, a condition that is probably associated with reduced expression of transcription factors belonging to the nuclear receptor family, such as peroxisome proliferator-activated receptors (PPARs) and their target genes, which regulate glucose homeostasis, lipid metabolism and inflammation [51,52]. However, both the aqueous and ethanolic extracts, regardless of dose, were unable to favor glucose metabolism in the animals fed a high-fat diet, thus promoting hyperglycemia and decreased beta cell functional activity, which is associated with an intense effect under oxidative stress [53].

Furthermore, a high-fat diet can modify oxygen metabolism because fatty acid molecules with double bonds are vulnerable to oxidative reactions that can lead to lipid peroxidation and, consequently, to oxidative stress [54].

Another fact, related to changes in glycemic metabolism and insulin resistance, is the accumulation of adipose tissue, which influences the expression of several proteins, cytokines and other molecules that are involved in several physiological or pathological processes [55]. In addition, the accumulation of adipose tissue favors an increase in the expression of tumor necrosis-alpha (TNF-α), monocyte chemotactic protein (MCP-1/CCL-2) and interleukin-6 (IL-6) by monocytes and macrophages [55]. These have a pro-inflammatory effect, and are directly related to the development of insulin resistance (IR) [18,56].

In the present study, the groups supplemented with *G. gardneriana* showed high levels of MCP-1, a pro-inflammatory cytokine, a condition that would explain the results in relation to the lipid and glycemic profile found above.

In contrast, interleukin 10 (IL-10) is a cytokine that plays a critical role, has potent anti-inflammatory properties and is produced by M2 macrophages in adipose tissue, with its main functions being to regulate the immune system and significantly inhibit the expression/synthesis of pro-inflammatory cytokines or adipokines via negative counter-regulation [57]. In the present study, a significant reduction in this cytokine was observed, supporting the hypothesis of the potential pro-inflammatory effect of MCP-1.

In studies that induced obesity in rodents, a reduction of plasmatic glutathione dismutase and superoxide dismutase, which are markers of antioxidant enzymes, was observed, associated with high levels of plasmatic TNF-α and IL-6 as pro-inflammatory markers. Therefore, it is noted that in the model of this work, when providing a hyperlipidic diet, the metabolic and structural abnormalities mimicked human obesity [44,58].

Similarly, insulin resistance generated in adipose tissue causes lipolysis to occur, increasing the release of free fatty acids into the circulation, and consequently elevates very low density lipoprotein (VLDL) concentrations and triglyceride synthesis in the adipose tissue. The accumulation of fat in the liver causes a decrease in liver function [59]. In addition, these conditions also contribute to the appearance of alterations in the serum lipid profile, such as high triglycerides, a decrease in high-density lipoprotein (HDL) and an increase in low-density lipoprotein (LDL), favoring the onset of dyslipidemia [60].

In this sense, the study of medicinal plants in the treatment of non-communicable chronic diseases has aroused the interest of researchers, with a view to studying the benefits of medicinal plants in the discovery of drugs for the treatment of diseases, based on different isolated bioactive compounds [61]. As the molecular control of the components found in medicinal plants is understood, it is possible to observe potential benefits in the prevention and treatment of current diseases [62].

Medicinal plants and their constituents may represent promising candidates for the treatment of obesity. Several medicinal plants and their active constituents show beneficial anti-obesity effects in vivo. Of all drugs available in therapy, around 25–30% are produced from natural products (plants, microorganisms, and animals) or are derived from these products [63]. On the other hand, the use of plants to counteract and/or prevent obesity is still poorly studied [64].

## 5. Conclusions

In the study, it was possible to verify that the aqueous extract of *G. gardneriana* at a dose of 200 mg/kg had a positive impact on food intake and HDL-c concentrations. The other groups, and even the HF AQ 200 group, did not show antioxidant or anti-inflammatory effects, and did not prevent weight gain, adiposity or changes in the lipid or glycemic profile.

In conclusion, under the experimental conditions, *G. gardneriana* did not prove to be a potential resource for the prevention of obesity and metabolic alterations; thus, more research is required to investigate the possible effects using different models, doses and time of investigation to determine the relationship between the phytochemical properties already elucidated in the literature and their medicinal potentials. A different behavior was obtained from that described in the literature with regard to the medicinal potential of Garcinia species.
